# Fear of falling is associated with physical functioning, activity performance, and participation in individuals with multiple sclerosis

**DOI:** 10.1002/pmrj.70131

**Published:** 2026-04-15

**Authors:** Libak Abou, Jeeyeon Kim, Chloe McCloskey, Cory Wernimont, Anna L. Kratz

**Affiliations:** ^1^ Department of Physical Medicine & Rehabilitation, Michigan Medicine University of Michigan Ann Arbor Michigan USA; ^2^ VA Long Beach Health Care System Long Beach California USA

## Abstract

**Background:**

Fear of falling (FoF) is a common concern in individuals with multiple sclerosis (MS). Understanding whether and how FoF affects activities and participation is crucial for developing effective rehabilitation programs in individuals with MS.

**Objective:**

To examine the relationship between measures of FoF (degree of FoF and avoidance behavior due to FoF) and measures of activities and participation in ambulatory individuals with MS. We also aim to investigate whether avoidance behavior due to FoF is relevant beyond the effect of degree of FoF.

**Design:**

Cross‐sectional survey‐based design.

**Setting:**

Community.

**Participants:**

A convenience sample of 531 community‐dwelling ambulatory individuals with MS.

**Interventions:**

N/A.

**Main Outcome Measures:**

Activities measures included Patient‐Reported Outcomes Measurement Information System (PROMIS) physical functioning and self‐efficacy to manage activities of daily living (ADLs). Measures of participation included PROMIS ability to participate in social roles and activities (SRAs) and Community Participation Indicators–Control subscale.

**Results:**

After controlling for age, gender, duration of MS, disability level, history of previous falls, and MS subtype, hierarchical linear regression analyses indicated participants with moderate and high FoF showed lower physical functioning and ability to participate in SRAs (*B* = −4.75 to −10.81, *p* < .01) compared to those with low FoF. Participants with high FoF also had reduced self‐efficacy in managing ADLs (*B* = −6.29, *p* < .01) and less control over community participation (*B* = 6.77, *p* < .05) compared to those with low FoF. Avoidance behavior due to FoF was associated with lower self‐efficacy to manage ADLs (*Β* = −1.59, *p* < .01), ability to participate in SRAs (*Β* = −2.62, *p* < .01), and control over community participation (*Β* = 3.34, *p* < .01).

**Conclusions:**

FoF is a significant predictor of activities performance and participation in individuals with MS. Our findings highlight the importance of assessing avoidance behavior due to FoF to inform rehabilitation approaches aimed at improving the participation and community integration of individuals with MS.

## INTRODUCTION

Multiple sclerosis (MS), a common chronic neurodegenerative disease,^1^ is characterized by gait and balance impairments^2^ that contribute to frequent falls and fear of falling (FoF).^3^, ^4^, ^5^, ^6^, ^7^ Over 50% of individuals with MS experience at least one fall within 6 months.^4^, ^8^, ^9^ Falls can have devastating consequences, including physical injuries,^9^, ^10^, ^11^ reduced independence,^9^, ^12^ and FoF.^13^, ^14^ They can also have a significant impact on overall health, well‐being, and quality of life,^15^, ^16^, ^17^ while also increasing burden on families, health care costs, and mortality risk.^18^, ^19^ FoF, both as a cause and consequence of falling,^20^, ^21^ affects >85% of individuals with MS,^22^ including >70% of those without a history of falls.^9^, ^23^ Unfortunately, FoF often creates a cycle of dependence, physical inactivity, muscular and cognitive decline, increased fall risk, and worsening FoF.^3^, ^21^, ^24^, ^25^ Therefore, over the past decade, numerous studies have explored FoF in this population,^21^, ^22^, ^26^ aiming to better understand its impact on individuals with MS and to develop rehabilitation strategies that break the associated vicious cycle.

The evidence indicates that FoF has been linked to both physical and psychological dysfunctions as well as a higher degree of MS symptoms.^27^, ^28^, ^29^ A recent systematic review concluded that individuals who report higher levels of FoF often exhibit greater cognitive impairments, increased fatigue, sleep disturbances, depressive symptoms, walking deficits (eg, smaller steps, lower gait speed), worse balance, and reduced mobility.^21^ Also, studies have reported that individuals with MS who experience higher levels of FoF engage in less physical activity than those with lower levels, regardless of their history of falls.^30^, ^31^ These aforementioned studies clearly delineate the mechanisms through which FoF may affect the functionality and independence of individuals with MS. The International Classification of Functioning, Disability and Health model emphasizes the importance of understanding whether and how FoF affects activity and participation domains, as this is crucial for identifying the most effective rehabilitation approach to enhance individuals' independence, well‐being, and quality of life.^32^ In this context, Sung et al. found that among wheelchair users with MS, FoF negatively affects community participation and quality of life.^33^ However, to our knowledge, no study has quantified the relationship between FoF and measures of participation in ambulatory individuals with MS.

In addition, studies to date have primarily focused on assessing the degree of FoF as evaluated using the Falls Efficacy Scale International (FES‐I), rather than measures that focus on impact of FOF on activities of daily living (ADLs)^34^, ^35^ Focusing solely on the degree of FoF without assessing its impact on ADLs may limit clinicians' understanding of its consequences on individual's functionality and independence. Therefore, evaluating the effect of FoF on ADLs (eg, avoidance of some ADLs due to FoF) can provide a more comprehensive assessment to improve interventions aiming at enhancing the functional independence and well‐being of individuals with MS. The present study aims to fill these gaps by examining the associations between FoF and measures of activities and participation in individuals with MS. We also aim to examine whether avoidance behavior due to FoF provides any additional significant findings above and beyond the degree of FoF commonly assessed in MS research.

## METHODS

A nationwide web survey, approved by the University of Michigan Institutional Review Board, was conducted between March and June 2023. The study was deemed to meet federal and institutional criteria for exempt human subjects research. Data were collected through Research Electronic Data Capture, a Health Insurance Portability and Accountability Act compliant online research tool that allows for direct data entry by study participants.^36^ Individuals who completed the survey had the option to enter a raffle to win 1 of 20 $100 gift cards. Part of the data collected in this survey has been previously published.^7^, ^9^

### Study participants

Eligibility criteria included (1) individuals who self‐reported having MS diagnosis by their medical doctor (ie, neurologist, MS specialist, primary care provider) and (2) were 18 years old or older. Participants responded to a question about their use of disease‐modifying therapy. No other inclusion or exclusion criteria were applied in an effort to obtain data from a diverse sample of individuals with MS across the United States. Participants were recruited through (1) an existing registry at the University of Michigan of people with confirmed MS, (2) advertisement of the study survey link on www.UMHealthResearch.org, and (3) through a National MS Society listserv email that distributed the survey link nationwide.

### Outcome measures

#### Demographics and clinical data

Participants completed information on demographics including age, gender, and race. Information on clinical data including MS subtype, duration of MS, falling status in the past 6 months (Yes/No) as described in previous studies,^7^, ^9^ and Patient Determined Disease Steps (PDDS),^37^ a measure of disease severity, was collected.

#### Degree of fear of falling

The seven‐item FES‐I was used to measure the degree of FoF.^34^, ^35^ The FES‐I asks individuals to report their concern about falling when participating or if they were to participate in a variety of ADLs. Although the FES‐I assesses the level of concern about falling, it has been widely used as the “gold standard” to measure FoF in individuals with MS.^21^, ^22^, ^30^, ^31^ This scale has been validated for use in clinical setting and research among individuals with MS.^35^ Scores from all seven items are summed, with higher scores indicating greater FoF. Scores ranging from 7 to 8 indicate low FoF, 9–13 moderate FoF, and 14–28 high FoF.^38^

#### Avoidance behavior due to fear of falling

Participants were asked to respond “Yes” or “No” to the question: “Do you think your fear of falling has made you cut down on any activities that you used to do?”^39^ This single question has been widely used in the MS literature to assess the impact of FoF on ADLs.^9^, ^33^, ^39^

#### Physical functioning

Patient‐Reported Outcomes Measurement Information System (PROMIS) Physical Function with Mobility Aid Short Form was used to assess physical function.^40^ This valid and reliable self‐reported measure of eight items examines the ability of individuals to walk 25 feet on a level surface with or without support and the ability to perform a series of daily living activities including picking up clothing from the floor, bend down, and get on and off the toilet.^40^ For those who are able to walk 25 feet on a level surface, three additional items are assessed and accounted for the total score. Total raw scores were converted into T score metrics (mean = 50 ± 10 for the general population).^40^ Higher scores indicates higher level of physical function.^40^

#### Self‐efficacy to manage ADLs

PROMIS Self‐Efficacy for Managing Daily Activities Short Form, a valid and reliable measure of self‐efficacy in managing various ADLs, was used.^41^ This self‐reported measure of eight items examines the self‐efficacy of individuals to perform various ADLs that include household chores, lift and carry groceries, and running errands.^41^ Total raw scores were converted into T score metrics (mean = 50 ± 10 for the general population).^41^ Higher scores indicate higher level of self‐efficacy in managing various ADLs.^41^

#### Social participation

PROMIS Ability to Participate in Social Roles and Activities, a valid and reliable measure of social participation, was used.^42^ This self‐reported measure of six items examines the ability of individuals to participate in social roles and activities that include doing regular leisure activities with others, family activities, and usual work.^42^ Total raw scores were converted into T score metrics (mean = 50 ± 10 for the general population).^42^ Higher scores indicate higher social participation.^42^

#### Community participation

The Community Participation Indicators (CPI), a valid and reliable measure of participation enfranchisement among people with disabilities including MS, was used in this study.^43^, ^44^ The CPI consists of two distinct subscales: importance of participation and control over participation.^43^, ^44^ The importance of participation subscale assesses perceptions of being valued by the community and contributing to it, whereas the control over participation subscale assesses individual's perceived choice and control over community participation.^43^, ^44^ The control over participation subscale was preferred in this study because its 13 items specifically examine aspects of control and responsibility related to participation enfranchisement that may be influenced by FoF. Raw score for the control over participation subscale range from 13 to 65.^44^ These scores are normalized on a scale of 0 to 100.^44^ Lower scores indicate higher control over community participation.^43^

### Statistical analysis

Demographics and clinical characteristics of the sample were summarized as mean ± SD for continuous variables because of the normal distribution of the data and as frequency and proportion for categorical variables. Bivariate analyses were conducted to examine the associations between the predictors (FES‐I and avoidance behavior), as well as between each predictor and all outcomes (see Appendix A). Four separate hierarchical multivariable linear regression analyses were conducted to examine the relationship between predictors (degree of FoF and avoidance behavior due to FoF) and each outcome variable that included physical functioning, self‐efficacy to manage ADLs, social participation, and community participation. For each outcome, we constructed a model to examine the relative contributions of avoidance behavior due to FoF above and beyond degree of FoF, demographics, and clinical variables. Demographics and clinical variables including age, gender, years since diagnosis, PDDS, history of falls in the past 6 months, and MS subtype were treated as covariates in these analyzes. Based on their clinical relevance and routine availability in the care of people with MS, these covariates were selected to estimate the association between FoF and functional outcomes independent of demographics, disease severity, and prior falls. Variables were entered in blocks into the hierarchical models using a stepwise approach. For each outcome, covariates including age, gender, years since diagnosis, PDDS, history of falls in the past 6 months, and MS subtype were entered in Block 1, the degree of FoF (FES‐I) was entered in Block 2, and avoidance behavior was entered in Block 3. Variations in R^2^ between each step were estimated to identify significant predictors at each stage of the analysis and to examine the unique contribution of each variable set. All analyses were conducted using the categorical classifications of the degree of FoF (low, moderate, and high FoF).^38^ Before model building, assumptions of multiple regression, including normality of residuals (visual inspection of histograms and data plots), independence of the residuals (Durbin–Watson's test: values close to 2), linearity and homoscedasticity (plots of the standardized residuals against the standardized predicted values), no multicollinearity (variance inflation factor [VIF] < 5), and no influential outliers (Cook's distance < 1), were assessed and no violation was observed (See Appendix B for an example). All statistical analyses were two sided, with the significant level set at *p* < 0.05. Statistical analyses were conducted using IBM SPSS Statistics for Windows (version 28.0, SPSS Inc., Chicago, IL, USA).

## RESULTS

Given the secondary nature of the study, the sample size was based on the number of participants enrolled in the primary study. However, our estimate indicates that the sample size was sufficient to detect a significant incremental effect of avoidance behavior above and beyond the effect of FoF. Assuming an α of .05, 80% power, and an effect size of 0.02, with two primary predictors and six covariates, a minimum sample size of 423 participants was required. A total of 531 ambulatory individuals who indicated an MS diagnosis were included in this study. Participants with missing data were excluded from the analyses, and data were not imputed because only ~3% of the data were missing for some variables (eg, CPI control and PROMIS Physical Function), resulting in a sample size of *N* = 513 for some models. Given the low proportion of missing data, complete‐case analysis was deemed appropriate, and the missing data are unlikely to have meaningfully affected the findings. Descriptive statistics for the study sample are presented in Table 1. Briefly, participants mean age was 54.4 ± 12.5 years, 80% female, 83% White, and 55% with relapsing remitting MS. These characteristics align with the national demographics of individuals with MS.^45^ This sample included only ambulatory individuals with MS with PDDS ranging from 0 (normal) to 6 (bilateral support to ambulate).

**TABLE 1:** Demographics (*N* = 531).

Characteristics	*N*	%	Mean	SD
Age (y)	531		54.4	12.5
Duration of MS	531		15.1	10.7
Gender				
Female	426	80.2		
Male	105	19.8		
Race				
White	443	83.4		
Black or African American	60	11.3		
American Indian or Alaska Native	3	0.6		
Asian	6	1.1		
Native Hawaiian or other Pacific Islander	1	0.2		
Bi‐/multiracial	7	1.3		
Unknown	3	0.6		
Not reported	9	1.7		
MS subtype				
Relapsing remitting	294	55.4		
Secondary progressive	123	23.2		
Primary progressive	71	13.4		
Progressive relapsing	18	3.4		
Not sure	25	4.7		
Disease modifying therapy				
Yes	423	79.7		
No	107	20.2		
Not reported	1	0.2		
PDDS				
Normal (0)	25	4.7		
Mild disability (1)	59	11.1		
Moderate disability (2)	41	7.7		
Gait disability (3)	79	14.9		
Early cane (4)	101	19.0		
Late cane (5)	101	19.0		
Bilateral support (6)	125	23.5		
History of fall				
Yes	328	61.8		
No	203	38.2		
Impact of fear of falling				
Yes	353	66.5		
No	178	33.5		
FES‐I	531		15.84	4.92
Low concern	23	4.3		
Moderate concern	175	33.0		
High concern	333	62.7		
PROMIS physical function	513		38.64	8.07
PROMIS self‐efficacy manage ADL	531		41.41	6.41
Ability to participate	524		41.83	7.66
CPI control	513		40.41	11.74

Abbreviations: ADL, activities of daily living; CPI, community participation indicators; FES‐I, falls efficacy scale‐international; MS, multiple sclerosis; PDDS, patient determined disease steps; PROMIS, patient reported outcomes measurement information system.

### Relationship between FoF and physical functioning

The findings of the hierarchical regression analyses examining the relationship between measures of FoF and physical functioning are presented in Table 2. The results indicated a statistically significant association between degree of FoF and physical functioning. Participants who reported moderate and high FoF had 4.75 and 10.81 lower PROMIS physical function, respectively, compared to participants with low FoF. Avoidance behavior due to FoF was not associated with physical function, above and beyond the effect of the degree FoF.

**TABLE 2:** Hierarchical linear regression analysis predicting physical functioning based on FoF predictors (*N* = 513).

	Step	*B* (95% CI)	Δ*R* ^2^
Covariates	1		0.41**
Age		−0.03 (−0.08 to 0.02)	
Gender; Ref (female)		0.21 (−1.02 to 1.45)	
Duration of MS		0.02 (−0.04 to 0.07)	
PDDS		−1.73 (−2.07 to −1.38)	
Falls history; Ref (No)		−1.34 (2.40 to −0.29)	
MS subtype; Ref (RR)			
Secondary progressive		−0.35 (−1.70 to 1.00)	
Primary progressive		−0.65 (−2.27 to 0.97)	
Progressive relapsing		1.60 (−1.16 to 4.35)	
Not sure		1.03 (−1.29 to 3.34)	
FES‐I; Ref: Low FoF	2		0.13**
Moderate FoF		−4.75 (−7.30 to −2.20)*	
High FoF		−10.81 (−13.50 to −8.13)*	
Avoidance behavior; Ref = No	3		0.01
Yes		−0.53 (−1.63 to 0.57)	
Total model adjusted *R* ^2^	0.53		

*Note*: VIFs ranged from 1.04 to 1.75; higher VIFs values (6.07–7.25) were limited to dummy‐coded categorical variables. * indicates statistical significance. ** indicates statistically significant variation in R^2^ at *p* < .01.

Abbreviations: CI, confidence interval; FES‐I, falls efficacy scale‐international; FoF, fear of falling; MS, multiple sclerosis; PDDS, Patient Determined Disease Steps; RR, relapsing remitting; VIF, variance inflation factor; Δ*R* ^2^, variation in *R* ^2^.

### Relationship between FoF and self‐efficacy to manage ADLs

Measures of FoF were also associated with self‐efficacy to manage ADLs (Table 3). Participants who reported high degree of FoF had 6.29 lower PROMIS self‐efficacy to manage ADLs compared to participants with low degree of FoF. Above and beyond the effect of the degree of FoF, participants who reported avoidance behavior due to FoF had 1.59 lower PROMIS self‐efficacy to manage ADLs compared to participants who did not perceive that FoF affects their activities. This resulted in a statistically significant variation in R^2^ (See Table 3).

**TABLE 3:** Hierarchical linear regression analysis predicting PROMIS self‐efficacy to manage ADLs based on FoF predictors (*N* = 524).

	Step	*B* (95% CI)	Δ*R* ^2^
Covariates	1		0.32**
Age		−0.06 (−0.10 to −0.01)	
Gender; Ref (Female)		0.69 (−1.43 to −0.83)	
Duration of MS		0.05 (0.01 to 0.10)	
PDDS		−1.13 (−1.43 to −0.83)	
Falls history; Ref (No)		−1.70 (−2.61 to −0.79)	
MS subtype; Ref (RR)			
Secondary progressive		0.66 (−0.52 to 1.83)	
Primary progressive		0.90 (−0.49 to 2.69)	
Progressive relapsing		0.34 (−2.02 to 2.69)	
Not sure		1.04 (−0.99 to 3.07)	
FES‐I; Ref: Low FoF	2		0.10**
Moderate FoF		−2.18 (−4.38 to 0.02)	
High FoF		−6.29 (−8.61 to −3.97)*	
Avoidance behavior; Ref = No	3		0.01**
Yes		−1.59 (−2.55 to −0.64)*	
Total model adjusted *R* ^2^	0.42		

*Note*: VIFs ranged from 1.05 to 1.75; higher VIF values (6.03–7.24) were limited to dummy‐coded categorical variables. * indicates statistical significance. ** indicates statistically significant variation in *R* ^2^ at *p* < .01.

Abbreviations: ADLs, activities of daily living; CI, confidence interval; FES‐I, falls efficacy scale‐international; FoF, fear of falling; MS, multiple sclerosis; PDDS, Patient Determined Disease Steps; PROMIS, Patient‐Reported Outcomes Measurement Information System; RR, relapsing remitting; VIF, variance inflation factor; Δ*R* ^2^, variation in *R* ^2^.

### Relationship between FoF and social participation

The findings of the hierarchical regression analyses examining the relationship between FoF and social participation are presented in Table 4. The results indicated a statistically significant association between measures of FoF and social participation. Participants who reported higher and moderate FoF had 8.66 and 4.82 lower PROMIS ability to participate in social roles and activities, respectively, compared to participants with low FoF. Above and beyond the effect of the degree of FoF, participants who reported avoidance behavior due to FoF had 2.62 lower PROMIS ability to participate in social roles and activities compared to participants who did not perceive that FoF affects their activities. This resulted in a statistically significant variation in R^2^ (See Table 4).

**TABLE 4:** Hierarchical linear regression analysis predicting PROMIS Ability to Participate based on FoF predictors (*N* = 524).

	Step	B (95% CI)	ΔR^2^
Covariates	1		0.22**
Age		−0.03 (−0.09 to 0.03)	
Gender; Ref (female)		−0.32 (−1.72 to 1.07)	
Duration of MS		0.07 (0.01 to 0.14)	
PDDS		−1.07 (−1.46 to −0.67)	
Falls history; Ref (No)		−1.30 (−2.48 to −0.11)	
MS subtype; Ref (RR)			
Secondary progressive		0.68 (−0.84 to 2.20)	
Primary progressive		1.91 (0.09 to 3.73)	
Progressive relapsing		−0.80 (−3.93 to 2.33)	
Not sure		0.29 (−2.34 to 2.91)	
FES‐I; Ref: Low FoF	2		0.09**
Moderate FoF		−4.82 (−7.67 to −1.97)*	
High FoF		−8.66 (−11.67 to −5.66)*	
Avoidance behavior; Ref = No	3		0.02**
Yes		−2.62 (−3.86 to −1.38)*	
Total model adjusted *R* ^2^	0.32		

*Note*: VIFs ranged from 1.05 to 1.76; higher VIF values (5.95–7.18) were limited to dummy‐coded categorical variables. * indicates statistical significance. ** indicates statistically significant variation in *R* ^2^ at *p* < .01.

Abbreviations: CI, confidence interval; FoF, fear of falling; FES‐I, falls efficacy scale‐international; MS, multiple sclerosis; PDDS, Patient Determined Disease Steps; PROMIS, Patient‐Reported Outcomes Measurement Information System; RR, relapsing remitting; VIF, variance inflation factor; Δ*R* ^2^, variation in *R* ^2^.

### Relationship between FoF and community participation

Similarly, measures of FoF were associated with community participation measured with the CPI control (Table 5). Participants who reported high FoF had 6.77 higher CPI control (meaning lower control) compared to participants with low FoF. Above and beyond the effect of degree of FoF, participants who reported avoidance behavior due to FoF had 3.34 higher CPI control (meaning lower control) compared to those who did not perceive that FoF affects their activities. This resulted in a statistically significant variation in R^2^ (See Table 5).

**TABLE 5:** Hierarchical linear regression analysis predicting CPI control based on FoF predictors (*N* = 513).

	Step	B (95% CI)	ΔR^2^
Covariates	1		0.08**
Age		−0.08 (−0.18 to 0.02)	
Gender; Ref (Female)		0.04 (−2.41 to 2.49)	
Duration of MS		−0.04 (−0.15 to 0.08)	
PDDS		0.61 (−0.09 to 1.31)	
Falls history; Ref (No)		2.80 (0.70 to 4.89)	
MS subtype; Ref (RR)			
Secondary progressive		−1.05 (−3.74 to 1.63)	
Primary progressive		−1.41 (−4.61 to 1.79)	
Progressive relapsing		0.68 (−4.96 to 6.32)	
Not sure		0.17 (−4.43 to 4.77)	
FES‐I; Ref: low FoF	2		0.04**
Moderate FoF		2.97 (−2.10 to 8.03)	
High FoF		6.77 (1.43 to 12.12)*	
Avoidance behavior; Ref = No	3		0.02**
Yes		3.34 (1.16 to 5.53)*	
Total model adjusted *R* ^2^	0.11		

*Note*: VIFs ranged from 1.04 to 1.75; higher VIF values (6.08–7.28) were limited to dummy‐coded categorical variables. * indicates statistical significance. ** indicates statistically significant variation in *R* ^2^ at *p* < .01.

Abbreviations: CI, confidence interval; CPI, community participation indicator; FES‐I, falls efficacy scale‐international; FoF, fear of falling; MS, multiple sclerosis; PDDS, Patient Determined Disease Steps; RR, relapsing remitting; VIF, variance inflation factor; Δ*R* ^2^, variation in *R* ^2^.

## DISCUSSION

The main purpose of the study was to enhance our understanding of the relationship between measures of FoF and measures of activities and participation among individuals with MS. The present findings highlight that above and beyond the effects of common demographics, history of falls, and disability level, FoF is a significant predictor of activity limitations and restriction in participation in individuals MS. More important, this is the first study to report that assessing avoidance behavior due to FoF among individuals with MS may provide clinically relevant information beyond the commonly used assessment of degree of FoF (FES‐I). This additional insight is essential for tailoring rehabilitation approaches to enhance the functionality and participation of individuals with MS.

Our findings indicate that the degree of FoF is inversely associated with physical functioning, self‐efficacy to manage ADLs, and social and community participation. Specifically, individuals with a FES‐I score ≥ 14 (high concern) consistently demonstrated lower levels of functionality and participation in our analysis. Similar findings have been reported in older adults^46^ and populations with neurological conditions.^47^, ^48^ For example, Liu et al. reported that for community dwelling survivors of stroke, the levels of FoF and its impact on ADLs, rather than levels of walking endurance, were significant predictors of community reintegration.^46^ Also, Kalron et al. reported that FoF, not falls, affects leisure‐time physical activity in individuals with MS.^30^ Our findings confirm the importance of identifying individuals who may benefit most from interventions targeting FoF to enhance functionality, well‐being, and quality of life.^22^ Due to the ubiquitous nature of balance and gait impairments leading to FoF in individuals with MS, more frequent screening of this symptom and its impact on ADLs is highly suggested in clinical settings.

Above and beyond the effect of the degree of FoF, our findings also shed light on the importance of assessing avoidance behavior due to FoF on activities and participation in individuals with MS. Specifically, reporting avoidance behavior due to FoF was strongly associated with lower self‐efficacy to manage ADLs and social and community participation in our sample. This is the first study to examine whether avoidance behavior due to FoF is associated with rehabilitation outcomes in individuals with MS and therefore, direct comparison with previous findings was not possible. However, this relationship is well established in older adults^49^, ^50^ and individuals with other neurological diseases such as Parkinson's disease.^47^, ^51^ Our findings on the importance of assessing avoidance behavior due to FoF may be relevant to identify individuals with MS for whom FoF is truly problematic. For instance, a person may report high degree of FoF on a FES‐I item that asks about walking in places with crowds, but that high score does not reflect whether they actually do walk in crowded places or avoid that activity because of their FoF. Complementing the FES‐I with an assessment of avoidance behavior due to FoF would provide a clearer picture of the problem of FoF and help guide providers in reaching the goal of improving the functionality and participation of individuals with MS.

It is important to highlight that avoidance behavior was assessed in our study using a single question about whether FoF affects the performance of activities. This represents an important methodological limitation, as the wording of this single question is broad and may reflect reduced involvement in activities rather than complete avoidance of those activities. This single question also fails to provide detailed information about specific activities or groups of activities that are avoided due to FoF. Consequently, it offers very limited information for guiding rehabilitation strategies to address FoF in the context of an activity and enhance patient independence and well‐being. This may explain the small variation in R^2^ that resulted from the step 3 of the hierarchical regression models. As an outcome measure in clinical trials, this single‐item question would have limited measurement reliability, clinical utility, and sensitivity relative to a well‐developed, multi‐item patient reported outcome measure. The clinical relevance of assessing avoidance behavior due to FoF underscores the need for a multi‐item outcome measure to assess the specific groups of activities that are affected by FoF and need to be targeted in rehabilitation approaches. A potential outcome to be considered in future studies in individuals with MS is the fear of falling avoidance behavior, which has been validated for use in older adults with Parkison's disease but has not yet been specifically validated in the population with MS.^52^

## LIMITATIONS

This study has several limitations. The cross‐sectional design of the study consisting of a snapshot of data precludes any conclusions regarding causality between predictors (degree of FoF, avoidance behavior due to FoF) and outcomes (physical functioning, self‐efficacy to manage ADLs, and participation). The addition of “avoidance behavior due to FoF” to the hierarchical regression models after the FES‐I yielded ΔR^2^ from 1% to 2%. Although these variations may appear small, their translation in the daily functioning of persons with MS may be clinically relevant. This is because FOF can be either protective, helping to prevent falls, or maladaptive, leading to functional limitations. Current clinical measures of FoF capture the degree of FoF but do not clarify how much it actually influences behavior. Clinicians and researchers often assume that higher FoF leads to greater activity restriction or reduced rehabilitation engagement, yet the true impact on behavior remains uncertain. Evaluating avoidance behavior due to FoF using a well‐developed, multi‐item outcome measure may provide clinically significant insights for designing FoF interventions that enhance safe independence, well‐being, and quality of life of persons with MS. Due to the survey nature of the study and the inability to obtain confirmation through medical records, we are unable to confirm the demographics and clinical data (eg, MS subtype) presented in this study. Also, the findings presented in the study may be impacted by recall bias, which may hinder the accuracy of the data (eg, history of previous falls in the past 6 months). Another limitation is that, although the FES‐I is widely used as the gold standard for assessing FoF, this measure asks individuals about their “concern about falling.”^34^, ^35^ In light of ongoing research about differentiating FoF and concern about falling,^24^, ^53^ we suggest that future studies examine how and whether these two constructs differently affect activities and participation. Although FoF cutoff scores have been widely used in individuals with MS to categorize them into low, moderate, and high FoF,^22^, ^54^, ^55^ these cutoffs were originally established for older adults^38^ and have not been formally validated in the population with MS. This may have contributed to the imbalanced distribution observed across FoF categories, which may in turn help explain the wide confidence intervals observed in the analyses. Moreover, because these analyses were exploratory and involved multiple outcomes, *p* values should be interpreted cautiously, with greater emphasis placed on the consistency, direction, and magnitude of effects rather than on statistical significance alone. Finally, we did not examine whether other factors such as fatigue, level of physical activity, depression, and cognition influence the relationship between FoF and the outcomes examined in this study. For example, the debilitating effect of FoF in individuals with MS may increase levels of fatigue, which in turn can increase the extent of other symptoms.^56^ Future studies should explore the moderating effects of common MS symptoms on the relationships between FoF, falls, activities, and participation.

## CONCLUSIONS

In summary, this study highlights that individuals with MS with higher degree of FoF report lower physical functioning, self‐efficacy to manage ADLs, and social and community participation. Moreover, the findings indicate that the assessment of avoidance behavior due to FoF may provide clinically relevant information above and beyond degree of FoF, regarding activities performance and participation in individuals with MS. Although prospective studies are needed to examine the associations presented in this study, a muti‐item outcome measure to appropriately assess avoidance behavior in individuals with MS is warranted. These future efforts will be crucial to better understand how FoF affects activities and participation and will be important to successful development of rehabilitation approaches to enhance the independence, well‐being, and quality of life of individuals with MS.

## FUNDING INFORMATION

This study was supported by the Dan Barry Research Program pilot grant from the Department of Physical Medicine and Rehabilitation at the University of Michigan. LA was supported by Michigan Institute for Clinical & Health Research‐MICHR K12 program (K12TR004374) from the National Center for Advancing Translational Sciences.

## ETHICS STATEMENT

This study was conducted according to the Declaration of Helsinki. Informed consent was signed by all participants. Participants voluntarily confirmed their decision to participate in this study.

## DISCLOSURE

The authors declare no conflict of interest.

## Supporting information


**Appendix A.** Bivariate analyses examining the relationships between predictors and outcomes of the study.

**Appendix B.** Example of residual plots for the regression model predicting physical functioning.

**Appendix C.** SPSS syntax used for hierarchical linear regression model predicting physical function.

## Data Availability

The data that support the findings of this study are available from the corresponding author upon reasonable request.
