# Supplementary material for: Fear of falling is associated with physical functioning, activity performance, and participation in individuals with multiple sclerosis
Source: PM R. 2026 Apr 15;18(10):1194–203. doi: 10.1002/pmrj.70131 (PMC13686106; doi:10.1002/pmrj.70131)
Supplement: Supplementary file 1 [file PMRJ-18-1194-s001.docx]

| Variables | Age | Sex | Duration of MS | PDDS^a^ | Falls history | FESI^a^ | Avoidance behavior^b^ |
| --- | --- | --- | --- | --- | --- | --- | --- |
| PROMIS Physical Function | -0.22** | -0.06 | -0.05 | -0.46** | -0.29** | -0.49** | -0.25** |
| PROMIS Ability to Participate in Social Roles and Activities | -0.09* | -0.05 | 0.05 | -0.27** | -0.24** | -0.40** | -0.32** |
| PROMIS Self-Efficacy | -0.18** | -0.02 | 0.01 | -0.39** | -0.30** | -0.46** | -0.30** |
| Community Participation Indicators (CPI) | -0.08 | 0.01 | -0.09 | 0.11** | 0.20** | 0.26** | 0.23** |

**Appendix A.** Bivariate analyses examining the relationships between predictors and outcomes of the study

* indicates p <0.05; ** indicates p <0.01; ^a^Kendall's Tau was used; ^b^ The Kendall’s Tau correlation between avoidance behavior and FESI was = 0.35**

**Figure 1.** Grouped boxplots of FES-I distribution by avoidance status
