# Supplementary material for: Fear of falling is associated with physical functioning, activity performance, and participation in individuals with multiple sclerosis
Source: PM R. 2026 Apr 15;18(10):1194–203. doi: 10.1002/pmrj.70131 (PMC13686106; doi:10.1002/pmrj.70131)
Supplement: Supplementary file 2 [file PMRJ-18-1194-s002.docx]

**Appendix C. SPSS syntax used for hierarchical linear regression model predicting physical function**

REGRESSION

/MISSING LISTWISE

/STATISTICS COEFF OUTS CI(95) R ANOVA COLLIN TOL CHANGE

/CRITERIA=PIN(.05) POUT(.10)

/NOORIGIN

/DEPENDENT PF_with_MobilityAid_TScore

/METHOD=ENTER age sex ms_diagnosed_years pddsclass fall_1 Dum_MSsubtype_2 Dum_MSsubtype_3 Dum_MSsubtype_4 Dum_MSsubtype_5

/METHOD=ENTER Dum_FESI_Categorized_2 Dum_FESI_Categorized_3

/METHOD=ENTER Impact_CAF_Cat

/SCATTERPLOT=(*ZRESID ,*ZPRED)

/RESIDUALS HISTOGRAM(ZRESID) NORMPROB(ZRESID)

/CASEWISE PLOT(ZRESID) OUTLIERS(3).
