# Supplementary material for: Fear of falling is associated with physical functioning, activity performance, and participation in individuals with multiple sclerosis
Source: PM R. 2026 Apr 15;18(10):1194–203. doi: 10.1002/pmrj.70131 (PMC13686106; doi:10.1002/pmrj.70131)
Supplement: Supplementary file 3 [file PMRJ-18-1194-s003.docx]

**Appendix B.** Example of residual plots for the regression model predicting physical functioning.

**Multicollinearity Testing for all models**

All continuous and key predictors showed low VIF values (<1.76), indicating no multicollinearity. Moderately elevated VIF values (up to ~7.3) were observed only for dummy-coded categorical covariates, which is expected given their coding structure.
